# Environmental dust repelling from hydrophobic and hydrophilic surfaces under vibrational excitation

**DOI:** 10.1038/s41598-020-71356-5

**Published:** 2020-09-01

**Authors:** Abba Abdulhamid Abubakar, Bekir Sami Yilbas, Hussain Al-Qahtani, Ammar Alzaydi, Sharif Alhelou

**Affiliations:** 1 Mechanical Engineering Department, KFUPM, Dhahran, 31261 Saudi Arabia; 2 Center of Research Excellence in Renewable Energy (CoRE-RE), KFUPM, Dhahran, 31261 Saudi Arabia; 3 Senior Researcher at K.A. CARE Energy Research and Innovation Center at Dhahran, Dhahran, Saudi Arabia

**Keywords:** Engineering, Mechanical engineering, Nanoscale materials, Synthesis and processing

## Abstract

Mitigation of environmental dust from surfaces becomes one of the challenges for maintaining the optical characteristics of surfaces. Dust repelling from hydrophobic and hydrophilic surfaces under vibrational excitation is investigated and the percentage of dust repelled from surfaces is evaluated. The characteristics of the dust particles are examined and dust adhesion on surfaces under molecular forces (van der Walls) is explored. High speed recording system is utilized to monitor dust repelling from the surfaces. The dust residues, which are not repelled from the sample surfaces, are analyzed and the percentage of area coverage of the dust repelled from the surfaces is assessed. The repelling height of the dust is predicted analytically, and the findings are compared with the experimental data. Findings revealed that the analytical predictions of dust repelling height are in good agreement with the experimental data. Due to none-stoichiometric elemental compositions in the dust compounds, ionic forces are created while forming the cluster-like structures because of particle adhesion. The vibrational excitation repels dust from sample surfaces in the form of cluster-like structures. Dust repelled from hydrophobic surface results in a larger clean area on the hydrophobic surface (80% of total surface area) than that of the hydrophilic surface (20% of total surface area).

## Introduction

Environmental dust mitigation from surfaces becomes one of the challenges in solar energy devices such as photovoltaic panels. Minimization of optical losses of the incident solar radiation becomes crucial towards maintaining high device performance. This is particularly true for solar panels located in the near region of desert environments; in which case, regular dust storms elevate technical challenges for cost effective mitigating dust from panel surfaces. Several techniques are introduced to clean dusty surfaces and some of these include electrostatic repelling^1,2^, brushing^3^, water splashing^4^, air jet blowing^5^, droplet rolling/sliding^6^, etc. Although some of the techniques introduced for dust mitigation are effective, they may find limited applications due to scarcity of clean water or expensive air compression. However, creating an electrostatic screen on the surfaces for electrostatic dust repelling lowers the optical transmittance of surfaces and, particulalrly, greatly influences the photovoltaic panel efficiency. In addition, maintaining of the mechanical systems that provide air blowing and water jet splashing, such as rotating parts, becomes challenging in dusty environments. Alternatively, vibrating dusty surfaces with sonically induced vibrational excitation can be one of the possible candidates for dust removal from surfaces. Frequency and intensity of the sonically induced vibration play a major role for the flexural motion of dusty surface. However, the method is only effective for cleaning within limited frequency and amplitude range. At very high frequency or sound intensity, structural damage of the dusty surface and other accessories can possibly occur^7^. On the other hand, dust particles possess various elements and compounds such as Na, K, Ca, Mg, Fe, S, Cl, O^2^. Some of the elements form non-stoichiometric compounds in the dust particle, such as Na_x_Cl_y_, or Ca_x_Cl_y_, and non-stoichiometric compounds contribute to the charges of the dust particles. Hence, dust adhesion on surfaces under van der Walls forces becomes unavoidable. This causes the clustering of dust particles and modifies dust pinning on surfaces. Since elemental composition distribution of dust across the dusty surface is non-uniform, the charge distribution becomes nonuniform on the dusty surface. Although dust has various shapes and sizes, the surface texture of dust minimizes the contact area between the dust particles and the surfaces due to small area gaps at the interfaces. However, the charged forces still cause pinning of particles on surfaces. As the particle size increases, surface flatness enhances and the dust particle pinning force at the interface also increases. This yields strong pinning of the small dust particles on surfaces. Changing the wetting state of surfaces towards hydrophobic reduces the adhesive forces between the dust and surfaces^8^. This weakens pinning of the dust particles on the surfaces. Moreover, a sonically induced vibrational excitation of the dusty surface can possibly mitigate the dust particles from surfaces. Consequently, investigation of the sonically induced vibrating of dust particles on hydrophilic and hydrophobic surfaces towards dust mitigation becomes essential.

Considerable research studies have been carried out to examine the excitation of surfaces by sonic and ultrasonic waves through which cleaning of surfaces such as PV panel and membranes becomes possible. Sonic/ultrasonic excitations enable vibration of textured surfaces with frequencies at which adhesive forces for pinning particles weakens on surfaces. Initial acceleration of surface, under sonically/ultrasonically induced vibrational excitations, enables to accelerate the particles on surfaces. Density variation between the excited surface and the particles and constrained displacement of the substrate surface create impulsive-like forces, which can cause particle repelling from the vibrating surface. Depending on the pinning force of the particles on surfaces and acceleration of surface, some particles can slide rather than jumping/impulsion while repelling from surfaces. The mechanism of particle repelling through jumping/sliding can be adopted to mitigate dust particles from solar panels; hence, the resulting cleaning mechanism can be extended to excite the liquid film on the PV panel surface towards removing highly adhered materials (dried waste) from surfaces^9^. High intensity acoustic waves can also be used efficiently removing the dust particles from surfaces via generating acoustic agglomeration of the particles initiating the bulk dust removal^10^. Ultrasonic excitation allows the particle to migrate and reposition on surfaces via levitation, which is particularly true as the particles are located in a fluid as in electrostatic precipitators; hence, ultrasonic excitation becomes one of the alternative particle removal methods from electrode surfaces^11^. In addition, tandem frequency ultrasonic excitation can create microbubbles in fluids, which, in practice, can be utilized to clean membranes while minimizing fouling effects^12^. Moreover, a significant number of studies are carried out formulating sonic excitation and vibrational motion of solid plates. Some simplified model studies provide insight into the plate elastic behavior for various boundary conditions, for example, a plate vibration under sonic boom^13^. The vibrating plates also find many applications from small to large scales mitigations. One of the challenges is that optimum designing and configuring of a system for the efficient operation under high-intensity sonic and ultrasonic excitations in fluid media towards cleaning of surfaces^14^. Nevertheless, the use of sonic/ultrasonic methods for cleaning applications is still in progress. On the other hand, exploring the adhesion of solid particles and their removal from surfaces is also one of the interesting research fields for space and environmental applications. Removing lunar dust or dust from solar panel surfaces requires further studies on solid adhesion related to molecular attraction^15^. Lunar dust adhesion on panels and optical elements is a typical example of the strong adhesion of particles on surfaces. Using electrostatic waves, Lunar dust can be mitigated from surfaces^16^. In addition, the assessment of photoelectromagnetic negative feedback enables to assess molecular attractions between the curved surfaces, such as curved panel surfaces^15^. In addition, numerical study towards evaluating particle movement under sonic wave fields provides insight into the particle acceleration and adhesion on plane surfaces^17^.

Particle mitigation from surfaces remains critical for the sustainable operation of optical and solar energy devices. Although various methods were purposed previously to mitigate dust from surfaces, sonic induced vibrational excitation of the plate has not been explored extensively for dust removal from hydrophobic surfaces^10,16,17^. Since the adhesion of dust on hydrophobic surfaces is weak, the use of sonic wavefields to clean dusty hydrophobic surfaces becomes fruitful. In the present study, environmental dust mitigation from hydrophilic and hydrophobic surfaces is examined under the vibrational sonic excitations. An experimental rig is designed and built to evaluate the oscillation of hydrophobic and hydrophilic plates in terms of frequency and amplitude under different excitation frequencies. The motion of the dust particles is recorded using high speed video recording system. The influence of inclination angle of plates is considered to assess the influence of gravity on the repelling dust particles from the surfaces. Dust repelling height and velocity are formulated incorporating the force and the energy balance and predictions are compared with those obtained from the high speed recording system.

## Experimental

Glass samples (20 × 20 × 0.3 mm^3^) were used to hydrophobize the surfaces. Dip coating technique was used to deposit the functionalized nano-silica particles on the sample surfaces. The process of synthesizing the nano-silica particles was carried out in line with the early study^18^. Tetraethoxysilane (TEOS), octyltriethoxysilane (OTES), ethanol, and ammonium hydroxide were mixed. The mixture was prepared using 14.4 mL of ethanol, 1 mL of distilled water, and 25 mL of ammonium hydroxide via string over 20 min. The silane was included in the final solution at a molar ratio of 3:4 and the resulting resolution was further stirred for 11 h at room temperature. The wetting characteristics of the nano-silica particles deposited surface were evaluated. A goniometer (Kyowa, model DM 501) was used to measure the contact angle and its hysteresis in accordance with the early work^19^. Surface texture and adhesion of a dust particle on sample surfaces are evaluated incorporating the atomic force microscope (AFM/SPM) probe at friction mode.

**Figure 1:**
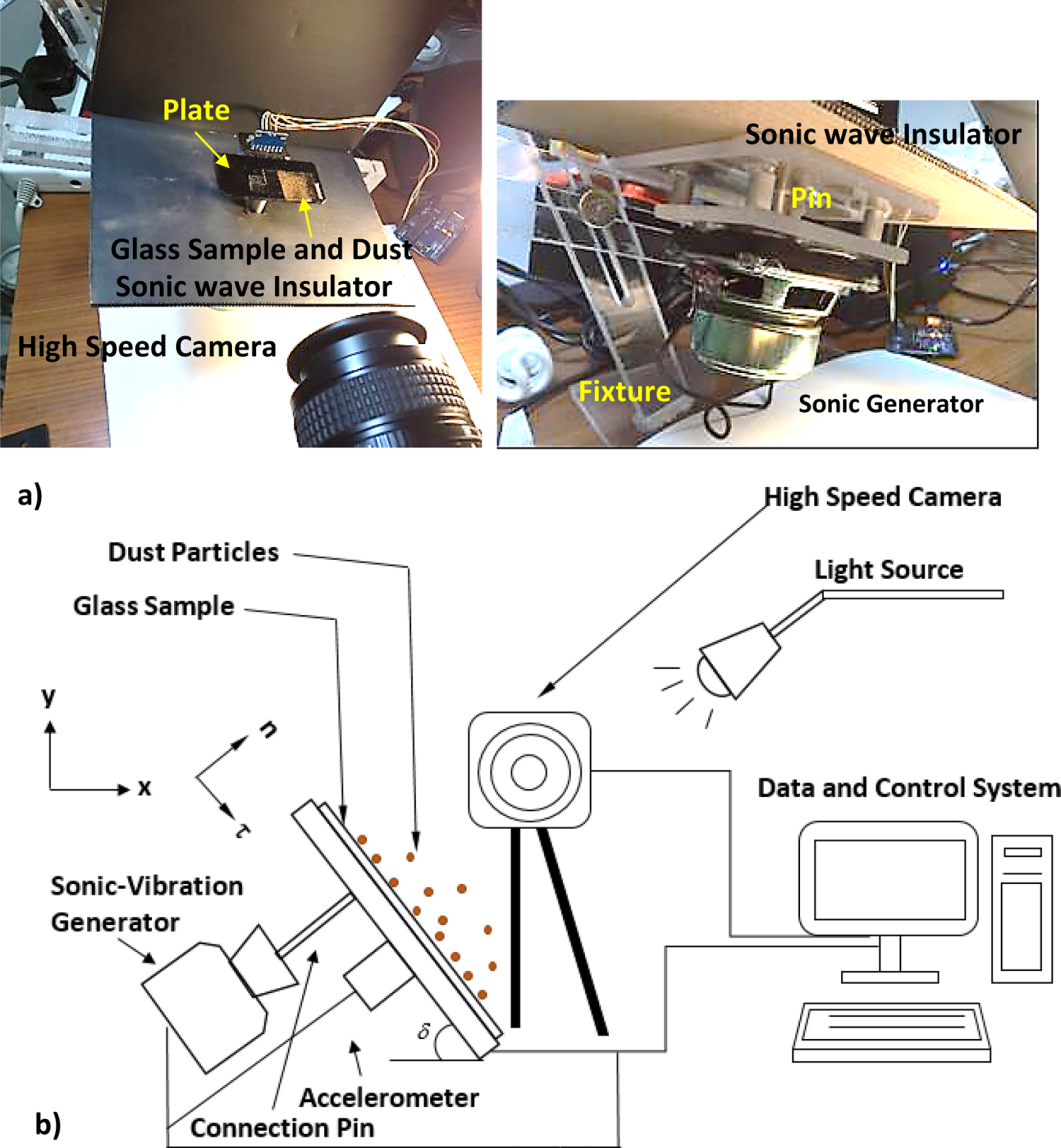
(**a**) Optical images of experimental setup, and (**b**) a schematic view of experiment.

Two-axes freedom table was designed and built (front and back inclinations with tilting reference to the horizontal position). The loudspeaker (made from Edifier Inc.) was operated at 9 V (DC) and 1.125 A and it was used to excite the table at different sonic frequencies. A metallic beam was placed between the pate bottom surface and the loudspeaker diaphragm where the sonic coil was located. The beam both ends were tightly fixed. Hence, the oscillation generated on the speaker coil was transmitted to the plate with the same frequency and displacement. Figure 1a shows optical images of the plate and loudspeaker system while Fig. 1b shows a schematic view of the experimental setup. The glass sample was placed on the plate with both ends were fixed. To avoid the excitation of the plate by the pressure (sonic) waves, which are generated by the loudspeaker, a sound reducer plate wafer was placed in between the loudspeaker and the plate. A fixture was designed and built allowing the assembly of the plate and the loudspeaker to tilt at various angles. This arrangement provided is used to examine the influence of gravity on the dynamic behavior of repelled dust from the sample surface. An accelerometer was used to ensure the same frequency of plate and speaker coil for several coil oscillation frequencies. Several tests were carried out to ensure the repeatability of the system at various excitation frequencies and amplitudes. A high speed recording system (Speed Sense 9,040) was utilized to monitor the sample and plate motions during the vibrational sonic excitations of the coil. The dust layer with a thickness of about 150 µm was laid on the sample surface. A high speed recording system was used to monitor the dust particles repelled from the sample surfaces during the vibrational sonic excitation. In addition, several tests were conducted to assess the maximum repelling height of the dust particles normal to the sample surface while altering the frequency of the loudspeaker coil oscillation. It was observed that the repelling height of the dust became maximum at 30 Hz and this frequency was set throughout the experiments. The high recording system was set to operate at 5,000 frames-per-second (fps) with the megapixel resolution of 1,280 × 800 and the pixel size of 14 µm × 14 µm. The repeatability of the recording data was evaluated via adopting the several repeats of the tests. The error estimated, based on the repeatability of the data, was about 3%. In addition, the measurement uncertainty (± *u*) was assessed after considering the range of values around the data measured (temporal repelled height of the dust particles) and the errors related to the displacements in recorded images (in terms of pixels). The confidence level of 95% with the standard deviation error distribution (range of a Gaussian) of 3% was obtained from the data recorded. The equation related to the standard uncertainty (*σ*_*u*_) can be written in the form of^18^:

1 $$ \sigma_{u} = \sqrt {\mathop \smallint \limits_{{x_{o} }}^{{x_{n} }} \left( {x - \mu_{e} } \right)^{2} p\left( x \right)dx} $$

where, *µ*_*e*_ corresponds to the mean/expected value of *x*, index *n* represents the number of data points, and *p*(*x*) is the probability distribution function. The probability distribution function for all possible displacement records of the clustered particles affecting the dust repelling velocity was primarily obtained from the instantaneous-correlation-plane. The probability distribution function was convolved with a suitable Gaussian-function for consistently estimating the probability distribution function diameter. Then, the standard uncertainty was determined to incorporate the least-squared Gaussian fitting. Later, the final evaluation was regulated (normalized) with the number of pixels, which contributed to the cross-correlation-peak. Moreover, the bias error of about 0.5 pixels was observed, which was related to the complexity of the sizing of very small peaks in the probability distribution function. The standard uncertainty was estimated at 3%.

Environmental dust was collected from surfaces of PV panels by incorporating the soft plastic-brushes and, later, dust was kept in vacuum-tight containers. Dust collected was analyzed using scanning electron microscopy and energy dispersive spectroscopy (JEOL 6,460), and X-ray diffraction (Bruker D8). The size distribution of dust was also evaluated via particle size analyzer (Microtrac).

## Results and discussion

Environmental dust mitigation from hydrophobic and hydrophilic surfaces under vibrational motion is investigated for the various tilt angle of the sample surfaces. A high speed recording system is used to assess dust particles' behavior under the vibrational sonic excitations. The dust particles are examined incorporating scanning and optical microscopes, energy dispersive spectroscopy, and X-ray diffraction. The adhesion of the dust particles on hydrophilic and hydrophobic surfaces is evaluated using an atomic force microscope.

**Figure 2:**
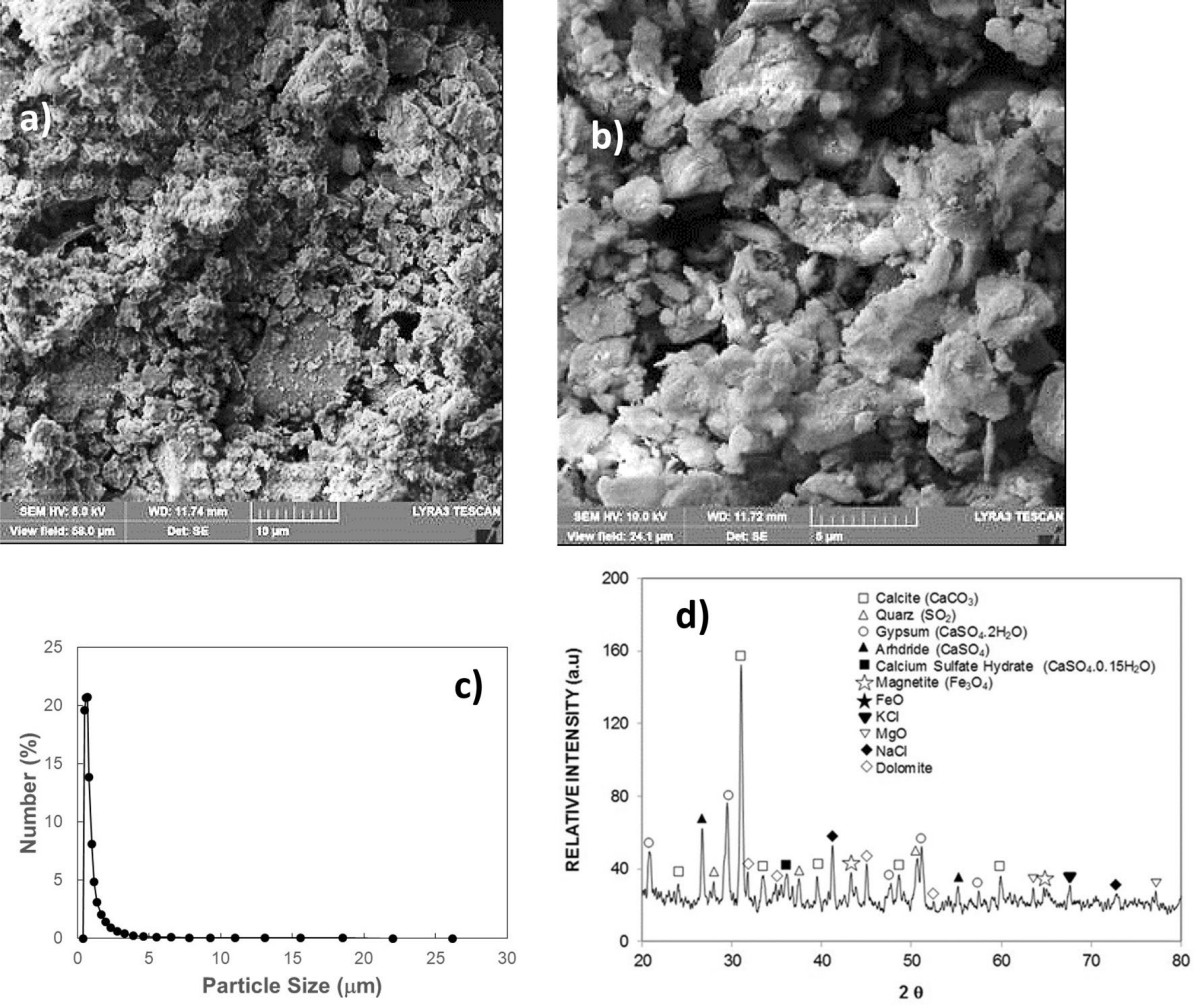
SEM micrograph of dust particles and dust particle distribution: (**a**) SEM micrograph of various sizes of dust, (**b**) SEM micrograph of various sizes dust, (**c**) size distribution of dust particles, and (**d**) X-ray diffractogram of dust.

**Figure 3:**
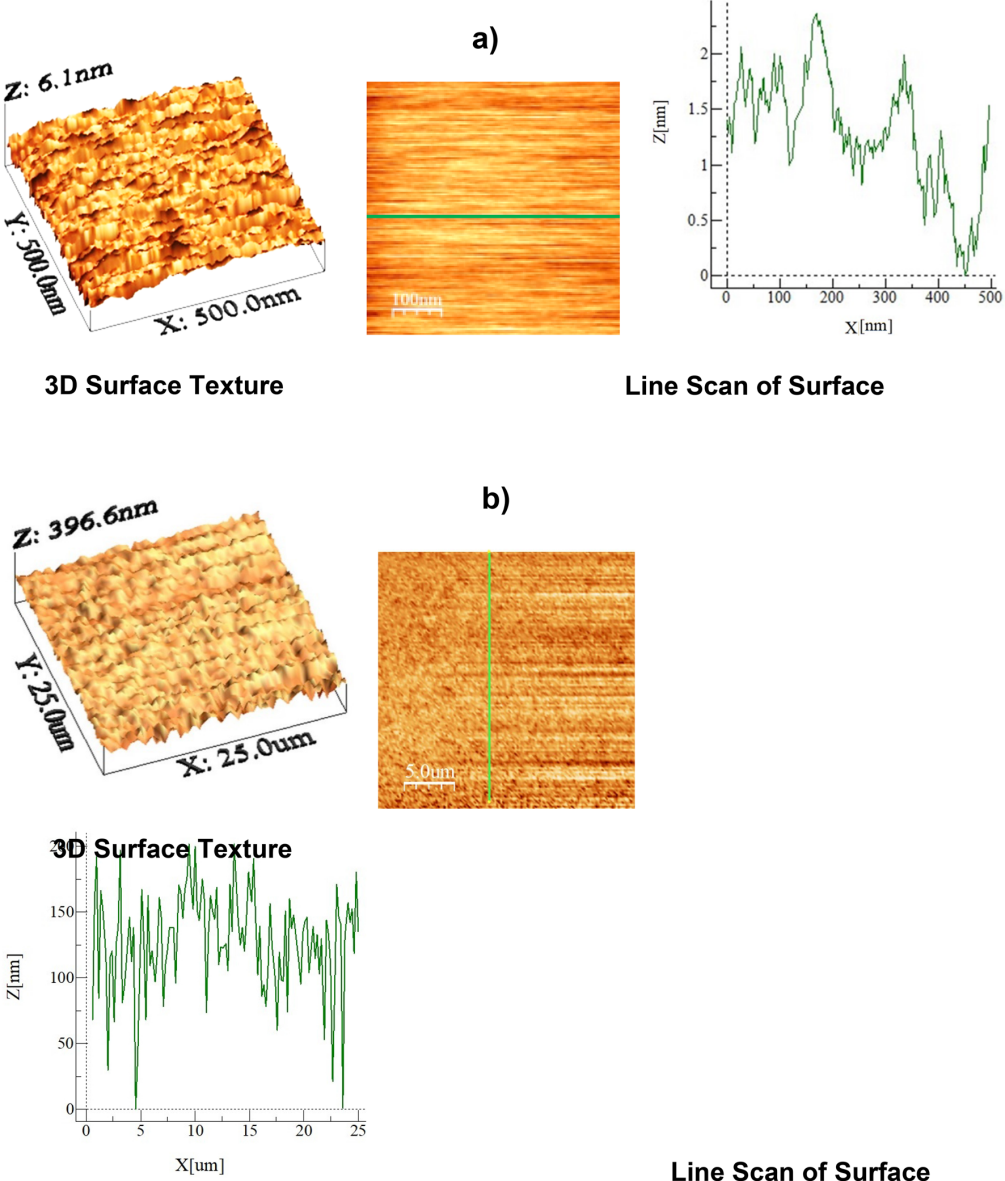
AFM images of plain and coated glass surfaces: (**a**) plain surface and (**b**) functionalized nano-silica particles deposited surface.

Figure 2a,b show SEM images of the dust while Fig. 2c provides the size distribution of the dust particles. The size and shape of dust alter considerably, and the average dust size is about 1.2 µm. The composition of dust changes slightly as the dust size changes (Table 1), i.e. dust size less than 1.2 µm has more oxygen content. X-ray diffraction of dust particles is depicted in Fig. 2d. The formation of NaCl and KCl compounds can be observed from an X-ray diffractogram. Since the elemental composition of these compounds does not satisfy the stoichiometric ratio, non- stoichiometric compounds of Na_x_Cl_y_ and K_m_Cl_n_ are formed in the dust particle. The non- stoichiometric ratio of elements in the compounds creates charges on the dust particles. Hence, small particles form cluster-like structures that are attached to the large dust particle surfaces (Fig. 3b). The surface free energy of the dust particles plays an important role in dust adhesion at the glass sample surfaces. To evaluate surface free energy of dust, the droplet contact angle technique is used in accordance with early study^21^. Three different liquids, namely water, glycerol, and ethylene glycol, are used to measure the contact angle of the droplet on the dust surface. Two methods are adopted to measure the contact angle of the liquids on dust. The Washburn technique^22^ is adopted firstly to measure the contact angle of fluids. In the second approach, small pellets are formed from the dust particles via brisk pressing and, later, the droplets are formed on the pellet surface. The contact angle measurements are carried out within ten seconds of the droplet formation to avoid the influence of droplet fluid diffusion into the pellet. Moreover, to evaluate the contact angle using the Washburn method, the dust particles are introduced into a glass tube with a 3 mm diameter, and the liquid bath is located under the tube. This arrangement allows the dust particles to draw-up the liquids under the capillary influence. The liquid mass increased per unit time in the tube is associated with the mass gain, which is formulated by Washburn as: $\frac{{\left( {\Delta m} \right)^{2} }}{\Delta t} = \frac{{C \cdot \rho^{2} \gamma cos\theta }}{\mu }$, here *Δm* represents the mass gain, *Δt* represents the duration for the mass gain (flow time), *C* is the capillary constant of dust, *ρ* is the fluid density, *θ* is the contact angle, *µ* is the fluid viscosity. To estimate the capillary constant (C), n-hexane is used in the fluid bath. This is because n-hexane results in zero contact angle (*θ*= 0). Several tests are carried out to ensure the repeatability of the experiments and data. Hence, the capillary constant for dust is estimated to be between 5.82 × 10^–16^ and 6.54 × 10^–16^ m^−5^. The discrepancy in the capillary constant can be related to the dust particles' shape and sizes, which can differ for each test. However, similar discrepancies are also reported in the literature when assessing the contact angle of powders^23^. The water droplet contact angle measured on the dust pellet is about is 38.2° ± 3° while it is estimated as 37.4° ± 3° from the Washburn method. Hence, both methods result in almost similar contact angle data. The surface free energy of dust can be obtained from the contact angle relations, i.e. $\gamma_{L} \left( {\cos \theta + 1} \right) = 2\sqrt {\gamma_{S}^{L} .\gamma_{L}^{L} } + 2\sqrt {\gamma_{S}^{ + } .\gamma_{L}^{ - } } + 2\sqrt {\gamma_{S}^{ - } .\gamma_{L}^{ + } }$^21,24,25^, here, subscripts *S* and *L* corresponds to the solid and the liquid segments, respectively, γ_S_ is the free energy of the solid surface, γ_SL_ is the interfacial solid–liquid free energy, γ_L_ is the surface tension of droplet liquid, *θ* is the measured contact angle, γ^+^ and—γare the electron acceptor and electron donor parameters of the acid–base component of the solid and liquid surface free energy, respectively. Table 2 gives the Lifshitz-van der Walls components and electron-donor parameters adopted in the calculations^21,24,25^. The surface free energy of the dust estimated from the contact angle method is about 111.5 ± 7.5 mJ/m^2^. To ensure the correct measurements, the tests were conducted eight times and the estimated error based on the repeatability of the data is about 7%. On the other hand, the dust adhesion on sample surfaces becomes important for vibrational repelling. Some model studies have been introduced for adhesion of particles on surfaces^26–29^. Because of the roughness of the dust particles and the sod surface, the equation introduced by Rabinovich et al.^29^ is adopted to estimate the dust adhesion on the sample surfaces. Hence, the adhesion force between the particle and the surface can be expressed as $F_{Ad} = \frac{{AR_{pd} }}{{12Z_{0}^{2} }}\left( {\frac{1}{{1 + \frac{{R_{pd} }}{{1.48r_{s} }}}} + \frac{1}{{\left( {1 + \frac{{1.48r_{s} }}{{Z_{0} }}} \right)^{2} }}} \right)$, here *A* corresponds to the Hamaker cstant (*A* = 0.48 × 10^–20^ J for SiO_2_^30^) and *Z*_*o*_ represents the particle spacing, which can be considered as the separation spacing between the dust particle and the sample surface, *R*_*pd*_ is the particle size, and *r*_*s*_ corresponds to the dust particle roughness parameter, which represents the ratio of the area of pillars on the dust surface over the projected area of dust surface. The roughness of the plain glass (hydrophilic) surface is about 1.2 nm (as obtained from AFM line scan, Fig. 3a) while that of the hydrophobized glass surface is about 156 nm (as determined from AFM line scan, Fig. 3b). The roughness parameter of the dust surface is determined from SEM images, which is within the range of 0.57—0.62. It is worth mentioning that several SEM images are produced to assess the roughness parameter, i.e. the variation in roughness parameter is because of the randomness in the texture of the dust particles as the dust particle size changes. Using the formula of Rabinovich et al.^29^, the adhesion force is estimated to be 4.4 × 10^–11^ N for 11 µm size dust particle after adopting the Hamaker constant A = 0.48 × 10^–20^ J, which is for silica^30^. Moreover, the adhesion force is also measured using the atomic force microscopy (AFM) probe in the contact mode. The AFM probe (tip) deflection is associated with the adhesion force in the form of $F_{add} = k\sigma \Delta V$^31^, where *k* corresponds to the spring constant of the tip (N/m) *σ* is the slope, which is estimated through the ratio of the tip displacement (*Δz*) over the probe voltage (*ΔV)*, i.e. *Δz*/*ΔV* in m/V) during the tip scanning on the surface where the dust particle is located. From the AFM probe calibration, the slope related to the tip deflection remains constant, which is *kσ* = 5.80275 × 10^–13^ N/mV. This gives rise to the adhesion force of 4.211 × 10^–11^ N for 11 µm size dust particles as located on the plain glass surface. The AFM measurement of the adhesion force for about 11 µm size dust on the glass surface is almost similar to that obtained from Rabinovich et al.^29^ equation (4.4 × 10^–11^ N). The adhesion force measurements are extended to include the dust particle located on the hydrophobic glass surface. The findings revealed that the adhesion force for the same size dust particle reduces to 2.234 × 10^–11^ N on the hydrophobic glass surface. Hence, the adhesion force for the dust particle on the hydrophobic surface reduces significantly. However, the adhesion force measurements are carried out for small size dust particles (~ 1.2 µm) while locating the small size dust particle on hydrophobic and hydrophilic sample surfaces. The force of adhesion obtained from the AFM probe for the hydrophilic surface is about 2.612 × 10^–10^ N and it becomes 6.321 × 10^–11^ N for the hydrophobic sample. Also, as the dust particles become small, the force of adhesion becomes high, i.e. as the dust particle size reduces from 11 to 1.2 µm, the force of adhesion increases almost three folds for the hydrophobic surface while this increase becomes almost six folds for the hydrophilic sample. The reduction in the force of adhesion on the hydrophobic surface is related to: i) reduced interfacial force (van der Waal forces) between the dust particle and hydrophobic surface because of the low surface free energy of the hydrophobized surface, and ii) increased surface roughness of the hydrophobized surface (156 nm as compared to 1.2 nm for plain glass surface), which reduces the contact area between the dust particle and the hydrophobized surface. Hence, the dust adhesion force on the hydrophobized glass surface becomes smaller than that of the hydrophilic glass surface. Moreover, the possible explanation for strong adhesion of the small dust particle (~ 1.2 µm) on hydrophobic and hydrophilic surfaces is associated with: i) forming of non-stoichiometric compounds (Na_x_Cl_y_ and K_m_Cl_n_, where x ≠ y and m ≠ n, Table 1) on small size dust particles, which results in ionic forces while increasing interfacial force between the small dust particle and the glass surface, and ii) as dust particle reduces (≤ 1.2 µm), in general, dust surface becomes smoother than the large size particles while increasing adhesion force between the particle as the glass surface because of the increased contact area. This finding is also consistent with the results of the early work^8^.

**Table 1:** Elemental composition of dust particles (wt%).

	Si	Ca	Na	S	Mg	K	Fe	Cl	O
Size ≥ 1.2 mμ	11.8	8.3	2.2	1.3	2.5	0.8	1.2	0.4	Balance
Size < 1.2 mμ	10.2	7.3	2.7	2.5	1.3	1.2	1.1	1.1	Balance
Dust residues	9.5	7.1	1.9	1.3	2.4	0.9	0.9	0.4	Balance

**Table 2:** Lifshitz-van der Walls components and electron-donor parameters used in the simulation^21,24,25^.

	γ_L_ (mJ/m^2^)	$\gamma_{L}^{L}$ (mJ/m^2^)	$\gamma_{L}^{ + }$ (mJ/m^2^)	s $\gamma_{L}^{ - }$ (mJ/m^2^)
Water	72.8	21.80	25.5	25.5
Glycerol	63.3	33.11	10.74	21.23
Ethylene glycol	48.2	31.09	6.59	11.16

The vibrational sonic excitation of the plate results in plate oscillation, which causes the acceleration of the dust particles on the plate surface. The force balance for a dust particle on the vibrationally excited inclined plate surface along the surface line (*τ*-axis) yields the acceleration of a particle, i.e.:

2 $$ \frac{{d^{2} h_{\tau } }}{{dt^{2} }} = g\sin \delta - \mu_{f} g\cos \delta - \frac{3\pi \mu D}{m}\frac{{dh_{\tau } }}{dt} $$

Similarly, a particle acceleration normal to the surface (*n*-axis) becomes:

3 $$ \frac{{d^{2} h_{n} }}{{dt^{2} }} = g\sin \delta - \frac{{C_{D} \rho \left( {\frac{{dh_{\tau } }}{dt}} \right)^{2} A}}{2m} - F_{ad} - \mu_{f} g\cos \delta . $$

where *m* is the dust particle mass, $h_{\tau }$ and $h_{n}$ are the dust particle relling height (displacement) along *τ* and *n*-axes, respectively ($h = \sqrt {h_{\tau }^{2} + h_{n}^{2} }$, *h* is the particle displacement from the plate surface), *t* is time, *g* is gravity, *C*_*D*_ is drag coefficient, *F*_*ad*_ is the adhesion force, $\mu_{f}$ is friction factor, $\delta$ is inclination angle of the plate. The formulation of Eqs. (2) and (3) are given in Appendix 1. Equations (2) and (3) are solved numerically to obtain the dust particle displacement on the plate surface.

Similarly, the particle velocity along the surface (*τ*-axis) is:

4 $$ v_{\tau } \left( t \right) = \frac{{mg\sin\delta - \mu_{f} mg\cos\delta }}{3\pi \mu D} - C_{1} me^{{ - \frac{3\pi \mu D}{m}t}} $$

**Figure 4:**
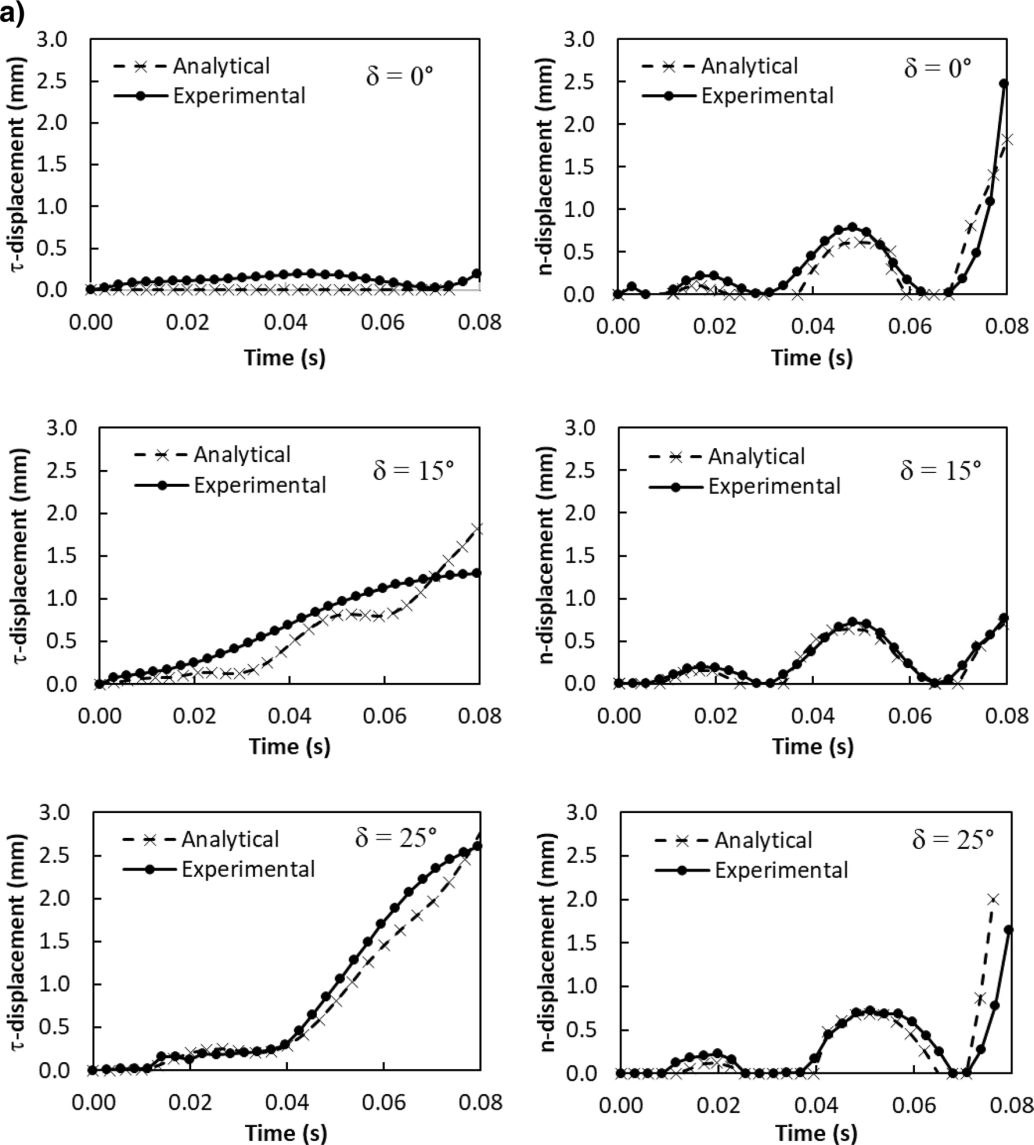

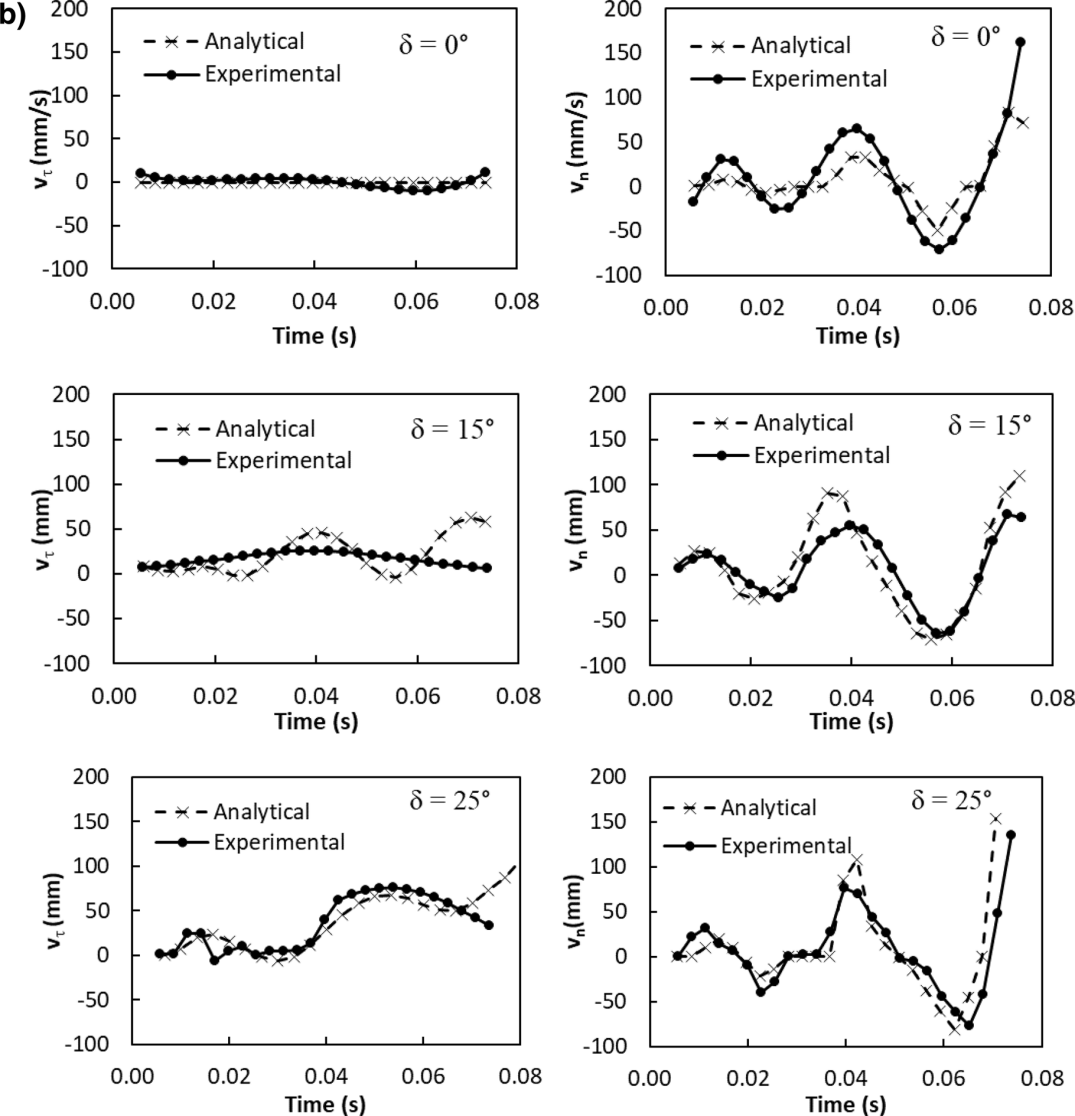
(**a**) Clustered dust particles displacement obtained from experiment and analytical solution along τ, and n axes on glass surface for different inclination angle of glass samples. (**b**) Clustered dust particles velocity obtained from experiment and analytical solution along τ, and n axes on glass surface at different inclination angle of glass samples.

**Figure 5:**
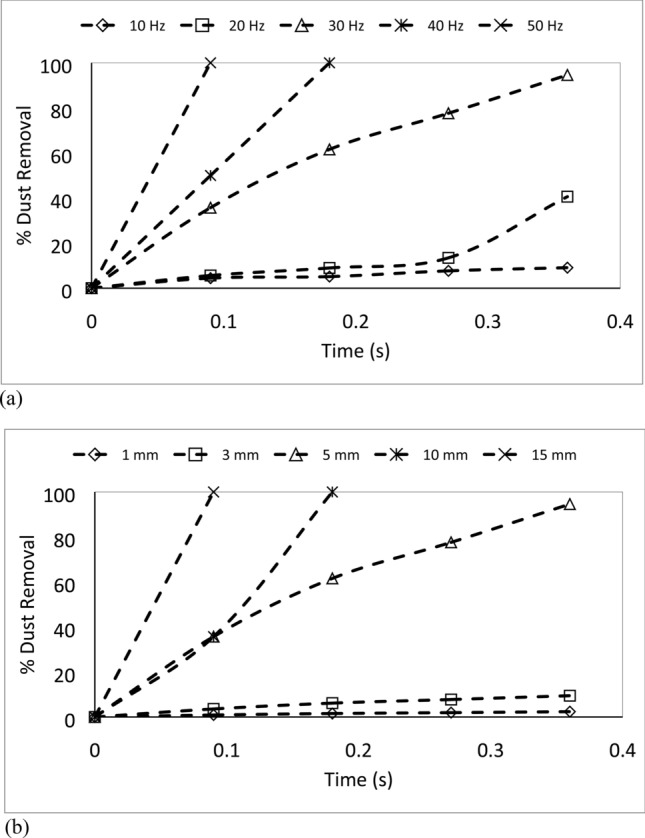
Variation of dust removed from hydrophobic surface with for 35°inclination angle of surface: (**a**) frequency and (**b**) amplitude of vibrating plate.

**Figure 6:**
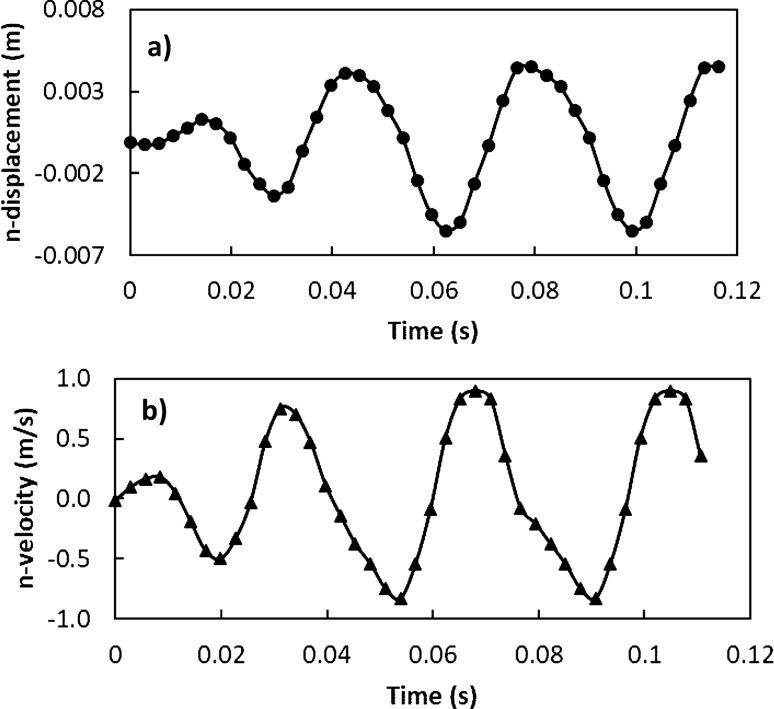
Plate displacement and velocity: (**a**) plate normal displacement under vibrational sonic excitation, and (**b**) plate normal velocity during excitation.

**Figure 7:**
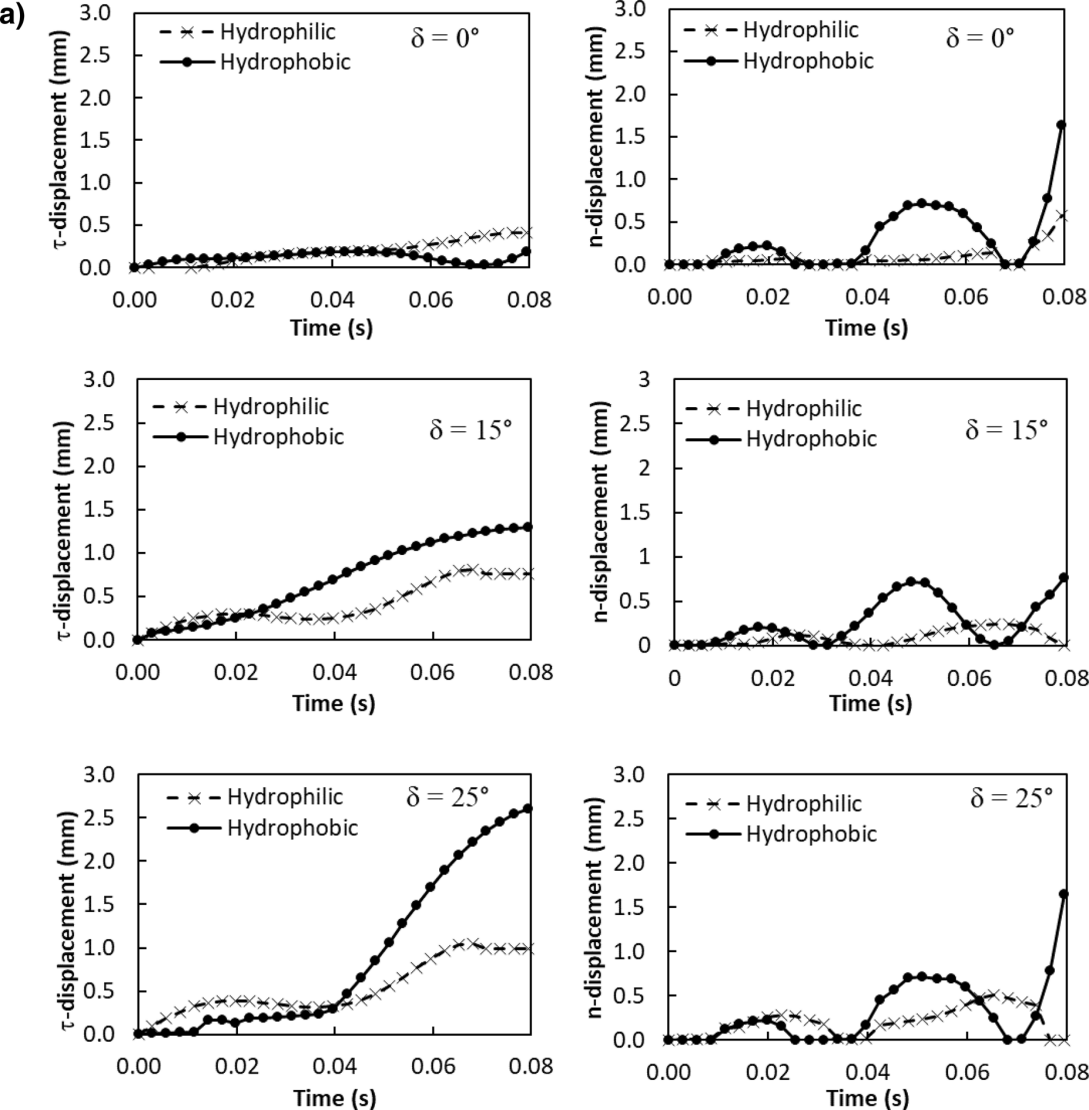

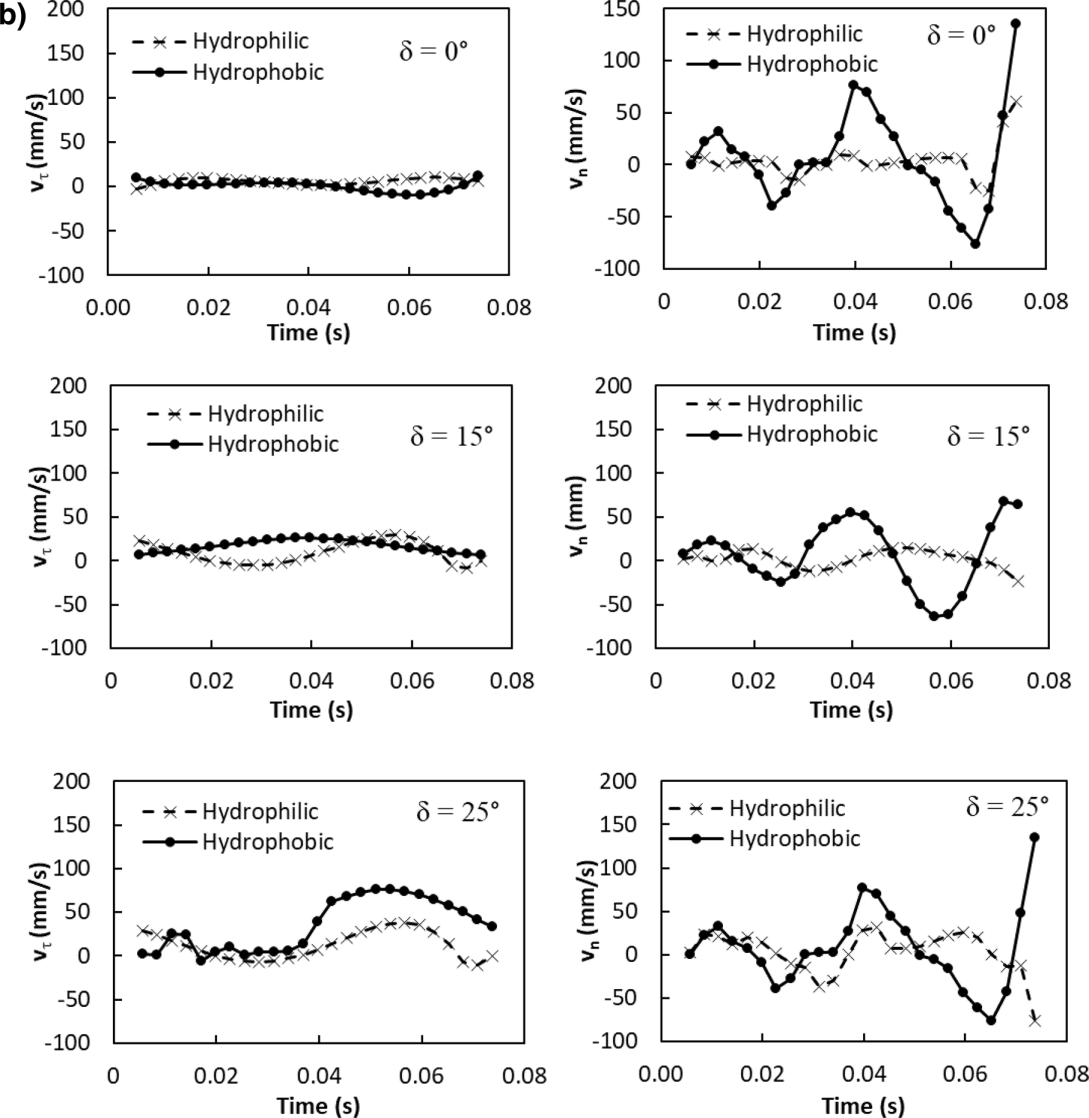
(**a**) Clustered dust particles displacement with time obtained from experiment along τ, and n axes on glass surface for hydrophilic and hydrophobic surfaces at different inclination angle of glass samples. (**b**) Clustered dust particles velocity with time obtained from experiment along τ, and n axes on glass surface for hydrophilic and hydrophobic surfaces at different inclination angle of glass samples.

The particle velocity along the surface (*n*-axis) is:

5 $$ v_{n} \left( t \right) = \frac{{ - mgcos\delta - F_{ad} }}{3\pi \mu D} - C_{3} me^{{ - \frac{3\pi \mu D}{m}t}} $$

where

$$ C_{1} = \frac{{\left( {g\sin \delta - \mu_{f} g\cos \delta } \right)m}}{3\pi \mu D} - v_{1} ;\quad C_{2} = \frac{{C_{1} m}}{3\pi \mu D};\quad C_{3} = \frac{{\left( { - g\cos \delta - \frac{{F_{ad} }}{m}} \right)m}}{3\pi \mu D} - v_{2} \quad {\text{and}}\quad C_{4} = \frac{{C_{3} m}}{3\pi \mu D} $$

here *v*_*τ*_*(t)* and *v*_*n*_*(t)* are the particle velocities along *τ* and *n*-axes ($v_{p} = \sqrt {v_{\tau }^{2} + v_{n}^{2} }$, *v*_*p*_ is the particle displacement from the plate surface), respectively, *t* is the time, and *F*_*ad*_ is the adhesion force of the particle on the surface. It is worthy to mention that the initial acceleration of the dust particles is evaluated from the vibration of the plate and formulation is provided in the Appendix. Figure 4a,b show temporal behavior of displacement and velocity of the dust particles obtained from the experiment and predicted from the analytical solution for 1.732 mm clustered dust particles and various inclination angles of the plate. It should be noted that τ and n-axes are laid in normal to the plate surface and the data obtained from the simulations and experiments are obtained according to the axes. Hence, the τ, n-axes simulation results are presented in Fig. 4a,b. In addition, the dust particles repelled from the surface form clustered-like structures because of the adhesion of the dust particles (among them, Fig. 4a). Since the glass sample is fixed on the oscillating plate subjected to the vibrational sonic excitation, the height of the clustered dust particles repelled from the plate surface is measured relative to the plate surface for all periods of plate oscillation. The displacement of the clustered dust particles along the τ-axis remains almost zero at the inclination angle of the plate is zero (*δ*= 0°) and small increase obtained from the experiment is associated with the experimental errors. As the inclination angle increases, the clustered dust particles displace along the τ-axis and the displacement enhances as the plate inclination angle increases further. Hence, the gravitational influence and vibrational sonic excitation of the clustered particles along with n and τ-axes enhance the dust displacement with an increasing inclination angle. It should be noted that the clustered particles repelled from the inclined surface under the vibrational sonic excitation follow a trajectory, which composes of *τ* and *n*-axes compounds. In the case of n-axis displacement of the clustered dust particles, the particle follows the oscillation of the plate under the vibrational sonic excitation. As time progresses, particle displacement along n-axis location enhances. The analytical findings are in good agreement with those obtained from the experiments. The displacement of the dust particles, which are shown in Fig. 4a, is obtained reference to the position of the glass sample surface. In this case, the plate undergoes an oscillatory motion due to vibrational sonic excitation with a frequency of the excitation of 30 Hz. It is worth noting that several tests are carried out at different frequencies and amplitudes of the vibrational excitations in order to find the optimum parameters for maximum dust removal rate, which bases on the total area cleaned from the dust on hydrophobic surface (Fig. 5). The sonic frequency of 30 Hz at 5 mm plate displacement amplitude is found to be favorable. This corresponds to peak plate velocity and acceleration of 0.0.9 m/s and 185.3 m/s^2^ respectively. At low frequencies (i.e. 0–20 Hz) dust removal was difficult due to low excitation energy and very short dust particle lifting height. While at high frequencies, (i.e. 40–50 Hz), the dust particles are repelled at a very high rate that could affect the integrity of the glass sample and other accessories. Hence, the experiments are carried out at this frequency. Figure 6a,b show the plate displacement and the plate velocity with time. The frequency of the plate remains the same as the vibrational excitation frequency (30 Hz, Fig. 4a). The maximum amplitude (displacement) of the plate increases in the early period (*t *≤ 0.042 s), which is related to the initial response of the plate to the vibrational excitation. However, as time progresses, the maximum amplitude of the plate oscillation becomes the same. Since both ends of the glass sample are fixed on the oscillating plate, the mode of oscillation of the glass sample remains the same as that of the plate. In the case of the clustered dust velocity (Fig. 4b), predictions agree well with the experimental data. In addition, the clustered dust particles almost follow the frequency of the plate. Hence, it reaches the maximum almost in the middle of the repelling height and reduces to zero as the clustered dust attains the maximum height. During the fall of repelled clustered dust particles, velocity becomes negative showing the falling clustered dust particles towards the sample surface. The velocity of the repelling dust increases slightly with the tilt angle of the plate. As the plate oscillation increases, the peak velocity of the clustered dust also increases. This behavior may be attributed to the reorientation of the clustered dust particles upon falling on the sample surface before excited by the vibrational motion of the plate, i.e. reorientation of the dust particles alters the adhesion of the clustered dust on the sample surface. Figure 7a,b show the dust particles displacement along τ, and n-axis for hydrophobic and hydrophilic surfaces at different inclination angles of the sample surface. The data presented in Fig. 7a,b are obtained from the high speed camera. The clustered dust displacement along τ-axis remains considerably small; however, the hydrophobic surface demonstrates large displacement along the n-axis from the surface. As the inclination angle of the surface increases, the clustered dust particles displacements in both τ and n-axes increase due to gravitational effect. The hydrophobic surface demonstrates a larger displacement of the clustered dust particles for all inclination angles as compared to that of the hydrophilic surface. This is mainly because of the pinning of the clustered dust particles, due to higher adhesion force, on the hydrophilic surface. As previously discussed by Quan et al.^8^, dust adhesion force on the hydrophobic surface is low because of lower surface energy associated with the highly rough (textured) surface. As the repelled dust falls back onto the sample surface, during the excitation period, the clustered dust adheres at the sample surface while reducing dust displacement on the sample surface under the vibrational excitation. In the case of the clustered dust particles velocity on the hydrophobic and hydrophilic surfaces (Fig. 7b), the clustered dust particles reach higher repelling velocity for the hydrophobic surface than the hydrophilic surface. The maximum repelling velocity for the hydrophobic surface becomes almost two-fold of the hydrophobic surface. The clustered dust behavior on the hydrophobic surface differs significantly in terms of repelling; hence, the use of the hydrophobic surface provides high velocity repelling of the clustered dust particles from the sample surfaces.

**Figure 8:**
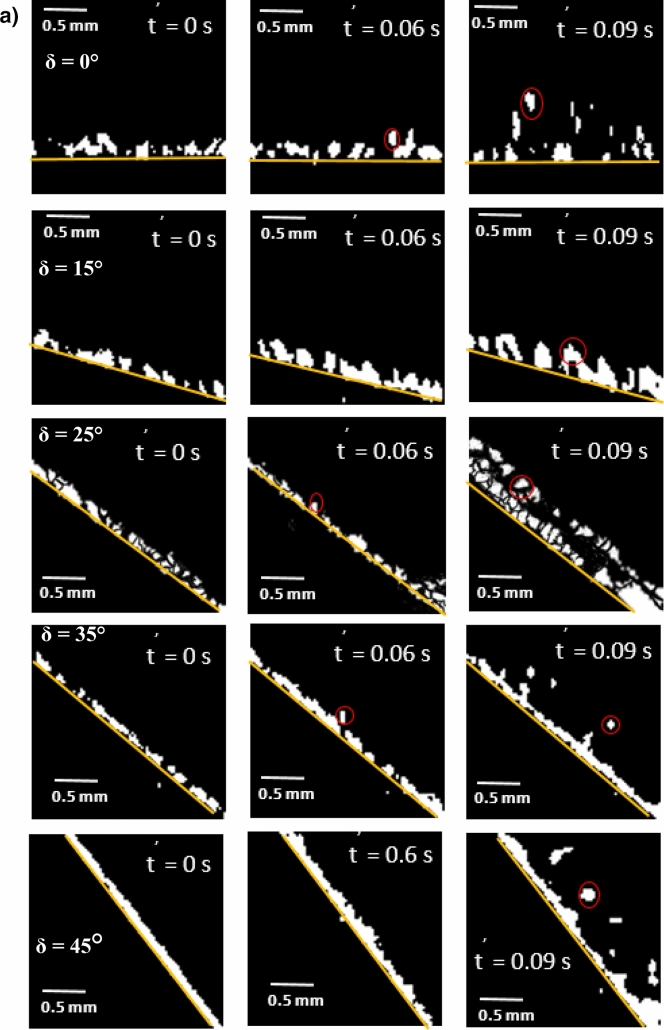

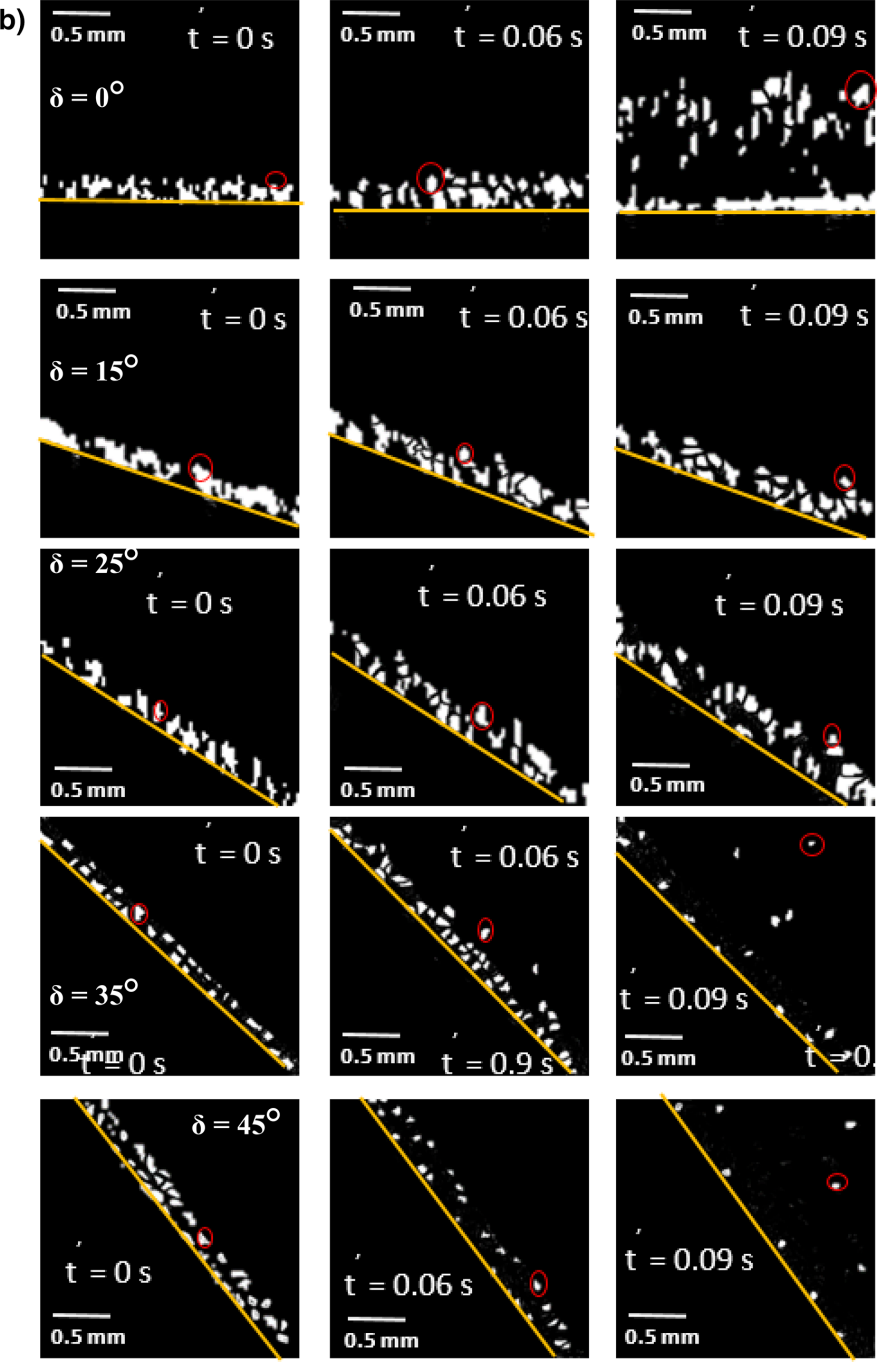
(**a**) Clustered dust particles repelled from hydrophilic glass surface at different inclination angle of the surface and times. Line shows sample surface. (**b**) Clustered dust particles repelled from hydrophobic glass surface at different inclination angle of the surface and times. Line shows sample surface.

**Figure 9:**
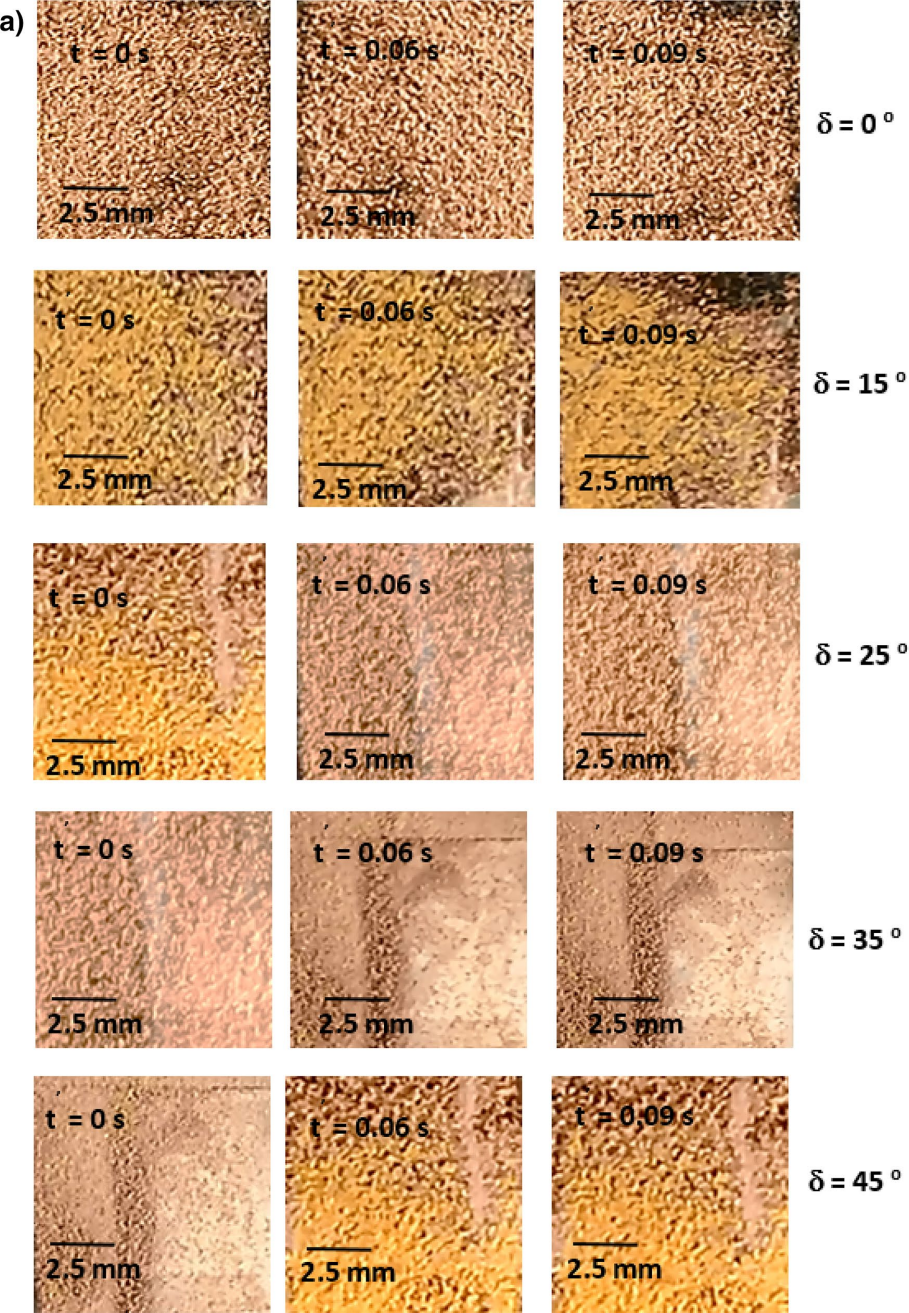

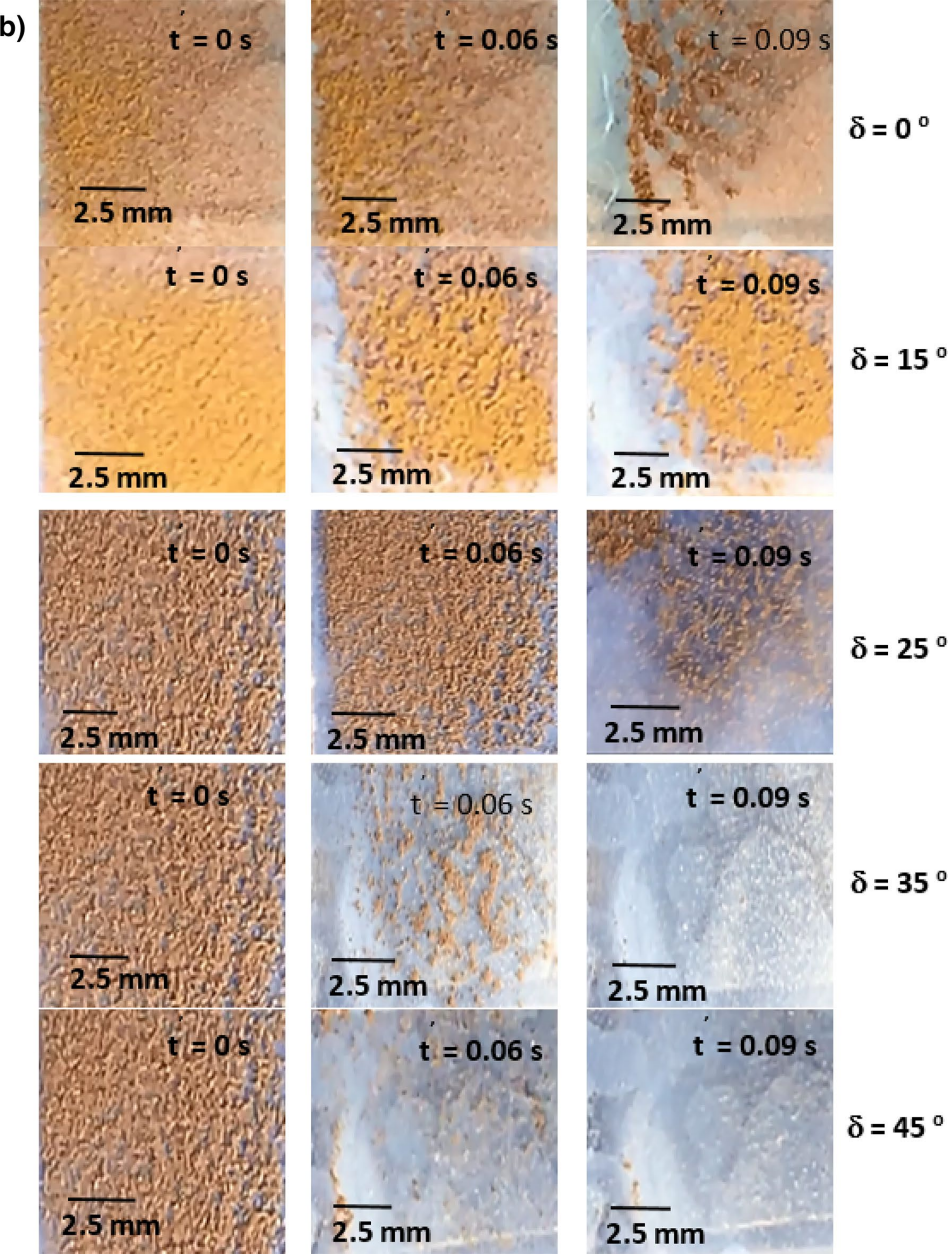
(**a**) Optical images of clustered dust particles on hydrophilic glass surface at different inclination angle of the surface and times. Line shows sample surface. (**b**) Optical images of clustered dust particles on hydrophobic glass surface at different inclination angle of the surface and times. Line shows sample surface.

**Figure 10:**
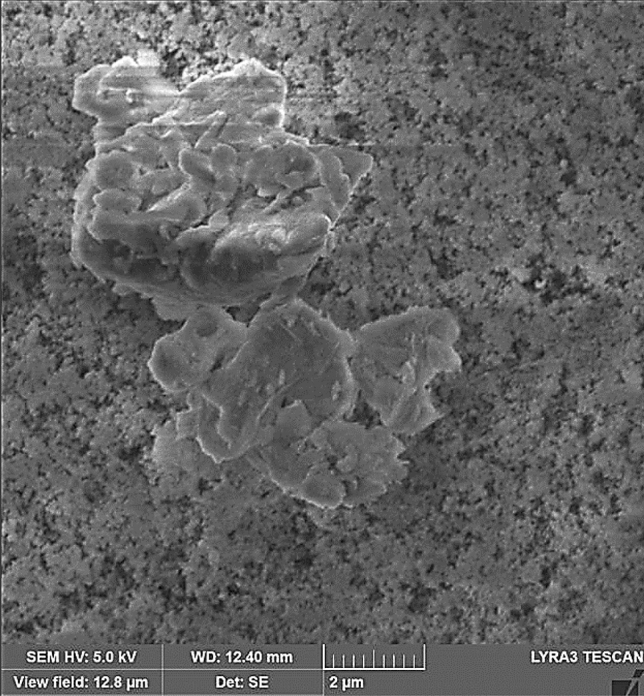
SEM micrograph of dust residues on hydrophobic glass surface after vibrational excitations.

**Figure 11:**
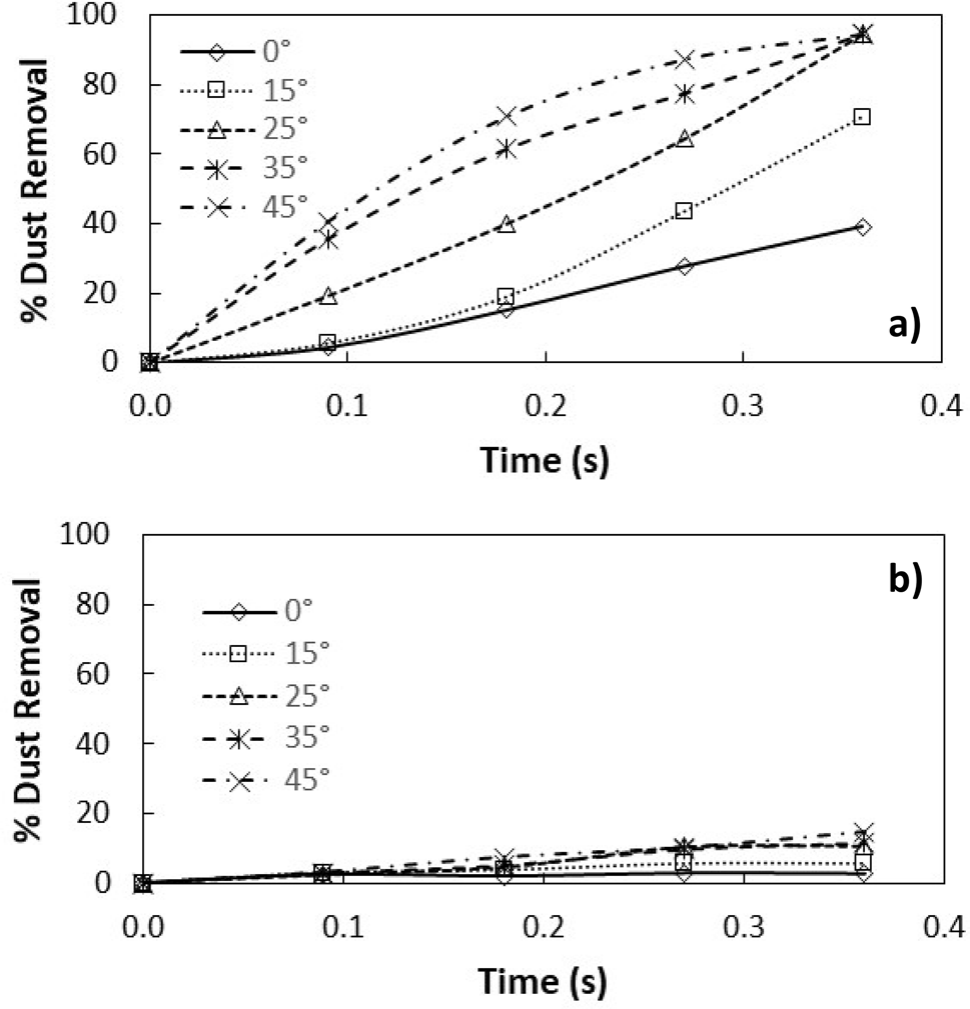
Percentage of dust removed from surfaces at various inclination angles of samples: (**a**) hydrophobic surface, and (**b**) hydrophilic surface.

Figure 8a,b show side views of the location of the clustered dust particles obtained from high speed recorded data for different inclination angles of the hydrophilic and hydrophobic glass samples at different times. The clustered dust particles reach the maximum height, which is normal to the sample surface, at about 0.09 s, which corresponds to 2.52 duration of the vibrational excitation. Since the frequency of vibrational excitation is 30 Hz, the corresponding cycle period is 0.0333 s. However, some clustered dust particles remain on the surface due to the high adhesion on the sample surface. In addition, some clustered dust particles, which are repelled from the surface falls back onto the sample surface during the vibrational excitation, which is particularly apparent for horizontally located glass sample (*δ* = 0). Hence, the external force generated on the dust particle by the plate motion due to vibrational excitation is not sufficient to overcome the adhesion of some clustered dust particles on the sample surface. Some of the dust particles, which may have fewer compounds with non-stoichiometric elemental composition, have weak adhesion on the glass sample surfaces. These dust particles can repel from the sample surface under the vibrational excitation of the plate. As the inclination angle increases, the clustered dust repelled from the sample surface increases; therefore, the dust particles pinning under the gravitational influence reduces by the value equal to the sign of the angle of the inclined plate. As comparing the dust clusters repelling from the hydrophobic and hydrophilic surfaces, the displacement height of the repelled dust clusters remains larger for the hydrophobic surface as compared to the hydrophilic surface. In addition, the number of dust particles remains on the sample surface during repelling becomes considerably less for the hydrophobic surface than that of the hydrophilic surface. This is mainly associated with the dust adhesion on the sample surface, which is almost three-fold higher for the hydrophilic surface than the hydrophobic surface. In order to assess the amount of dust residues on the glass samples surface, high speed optic camera is used to take the images of the dusty sample surfaces during vibrational excitation. Figure 9a,b show time frames of the high speed optical images of the top view of the sample surfaces with the presence of dust particles at different plate angles for hydrophilic and hydrophobic cases, respectively. The dust residues on the horizontally located sample surface are considerably larger than those of the inclined surfaces, which is true for hydrophilic and hydrophobic surfaces. However, the amount of dust residues on the surface remains considerably low for the hydrophobic surface, which is more apparent for the large inclination angle of the samples. The approximate weight of the clustered dust particles with a size of 200 µm is about 1.15 × 10^–7^ N and the repelling force of the same size of the clustered dust is about 1.44 × 10^–7^ N. Since the repelling force remains larger than the gravitational force for the clustered dust removal from the sample surface, the pinning force of the clustered dust, because of adhesion, becomes critically important for the dust residues, which are not repelled from the sample surfaces. Hence, as the pinning force, due to adhesion of the clustered dust on the sample surface, becomes larger than the repelling force than the dust remains on the sample surface. The influence of the clustered dust pinning can be observed via comparing the amount of the dust residues on the inclined surface of hydrophilic and hydrophobic samples. This situation can be observed from Fig. 9a,b, in which the top views of the hydrophilic and hydrophobic sample surfaces are shown. In addition, few of small dust particles remain on the sample surfaces after the excitations. Figure 10 shows SEM micrograph of small size dust residues on the hydrophobized glass surface. The dust resides have sharp edges/corners, which can anchor on the coated surface and can create a clustering effect while preventing dust repelling from the surface under the vibrational excitation. The percentage of the area where dust is removed via repelling from the sample surface is also determined. Figure 11a,b show the temporal behavior of the area percentage at different inclination angles of the sample for hydrophilic and hydrophobic sample surfaces, respectively. The area percentage represents the ratio of the surface area of the dust particles repelled (removed) over the total sample surface area. In general, the increase of the area percentage of dust removed from the sample surface follows almost linear behavior with time. The slope of the area percentage of dust removal from the surface increases as the inclination angle of the surface increases, which becomes more apparent for the hydrophobic sample surface. The area percentage of dust removed from the hydrophobic surface reaches almost 80% after 0.36 s of vibrational excitation. However, in the case of the hydrophilic surface, the percentage of dust removal from the sample surface remains significantly low, which becomes less than 20% after 0.36 s of the vibrational excitation for all inclination angles. Consequently, adhesion of dust particles first forms clustered-like structures on the surface and the repelling those clustered-like structures from the surface remains extremely difficult due to the adhesion of those structures onto the sample surfaces.

## Conclusion

Environmental dust mitigation from hydrophilic and hydrophobized glass surfaces is investigated under the influence of vibrational excitation. The influence of tilting angle of the sample surface on the dust removal rate is also examined. The percentage of dust repelled from samples are compared for hydrophilic and hydrophobic surfaces. Dust repelled from the sample surfaces is evaluated experimentally incorporating the high speed recording system. Dust repelling height is formulated analytically, and findings are compared with those obtained from the experiments. The findings revealed that the predictions of dust repelling height are in good agreement with those of the experimental data. Dust consists of various elements, which form non-stoichiometric compounds in the dust particles, i.e. some of the compounds formed do not satisfy the elemental stoichiometric ratio such as Na_x_Cl_y_ and K_m_Cl_n_ (x/y and m/n do not satisfy the stoichiometric ratio as evident from EDS data). This gives rise to charges on the dust particles and enhances the van der Walls forces while attracting the dust particles towards forming the clustered-like structures, which is more apparent for small size dust particles (≤ 1.2 µm). The vibrational excitation at 30 Hz is found to be the most effective exciting frequency for dust repelling from the hydrophobic and hydrophilic surfaces. The oscillation of samples exactly follows the frequency of the vibrational excitation of the sample holder (plate). Increasing the inclination angle of the samples enhances the repelling height of the dust clusters on the sample surfaces. In this case, reduced pinning force due to gravity is responsible for the increased vertical acceleration of the dust clusters from the sample surfaces. The velocity of the dust clusters is larger for the hydrophobic surface as compared to that of the hydrophilic surface, which is true for all inclination angles of the sample surface. The atomic force microscopy data reveals that the adhesion force for the dust particle on the hydrophobic surface remains significantly less than that of the hydrophilic surface. Hence, strong adhesion of the dust clusters on the hydrophilic sample surface lowers the repelling velocity. The dust clusters, which could not be repelled from the sample surfaces remain as residues. The dust residues cover relatively larger surface areas for the hydrophilic sample than that corresponding to the hydrophilic surface and almost 80% of the sample surface is cleaned via dust repelling from the hydrophobic surface at a sample inclination angle of 25° and more. However, the percentage of the area of the dust repelled from the surface remains less than 20% of the total area of the hydrophilic surface. Hence, hydrophobizing sample surfaces enhance the dust repelling from the surface, which is more apparent as the inclination angle of the surface increases. The present study gives insight behavior of dust removal from the hydrophobic and hydrophilic surfaces under the vibrational excitation and provides useful information for the environmental dust removal from surfaces.

## Supplementary information

Supplementary information
