# Supplementary material for: Environmental dust repelling from hydrophobic and hydrophilic surfaces under vibrational excitation
Source: Sci Rep. 2020 Sep 1;10:14346. doi: 10.1038/s41598-020-71356-5 (PMC7462990; doi:10.1038/s41598-020-71356-5)
Supplement: Supplementary file 1 — Supplementary information [file 41598_2020_71356_MOESM1_ESM.docx]

Supplementary information

**Environmental Dust Repelling from Hydrophobic and Hydrophilic Surfaces under Vibrational Excitation**

**Abba Abdulhamid Abubakar^1^ Bekir Sami Yilbas^1,2,3^ Hussain Al-Qahtani^1^ Ammar Alzaydi^1^ Sharif Alhelou^1^**

^1^Mechanical Engineering Department, KFUPM, Dhahran 31261, Saudi Arabia

^2^Center of Research Excellence in Renewable Energy (CoRE-RE), KFUPM, Dhahran 31261, Saudi Arabia

^3^Senior Researcher at K.A.CARE Energy Research & Innovation Center at Dhahran, Saudi Arabia

**Appendix 1: Formulation of a Particle Repelling from Surface**

The power input ($P_{in})$ to the speaker is expressed as a function of the current and voltage supplied to the speaker as:

$P_{in}=V\cdot I$ (A1)

here: $V$ is voltage, and $I$ is supplied current. The output power ($P_{out}$) acting on the diaphragm of the speaker is expressed as:

$P_{out}=\eta\cdot P_{in}$ (A2)

where: $\eta$ is efficiency of the loudspeaker. The efficiency of loudspeakers is usually low (i.e. around 0.5 – 2 %), because larger amount of energy is dissipated as heat in the electronic parts. Then, the average output power is radiated to the diaphragm causing a sinusoidal displacement. Thus, the average output power can be further expressed as the product of root-mean-square velocity of diaphragm ($\dot{x}_{rms}$) and force exerted ($F_{rms}$):

$P_{out}=\dot{x}_{rms}\cdot F_{rms}=\dot{x}_{rms}\cdot p_{rms}{\cdot A}_{s}$ (A3)

here: $p_{rms}$ is the root-mean-square pressure acting on diaphragm interior of surface area $A_{s}$ ^1^.

The pressure on the diaphragm can be related to the acoustic resistance of the surrounding medium ($R_{a}$) as:

$$p_{rms}=\dot{x}_{rms}\cdot R_{a}$$

 (A4)

The sound wave is often considered low when the frequency($f$) is less than the transition frequency${(f}_{t}=\frac{c_{0}}{\pi d_{d}\sqrt{2}})$. For low frequency sound, the acoustic resistance was previously found to be:

$R_{a}\approx\frac{\rho_{0}A_{s}\omega^{2}}{2\pi c_{0}}$ (A5)

where: $\rho_{0}$ is density of air, $\omega$ is angular frequency, and $c_{0}$ is speed of sound in air. Substituting Equations (A5) and (A4) into Equation (A3), the average sound power can be expressed as:

$P_{out}=\dot{x}_{rms}^{2}\cdot\omega^{2}\frac{A_{s}^{2}\rho_{0}}{2\pi c_{0}}$ (A6)

From Equation (A6), the root-mean square velocity of the diaphragm can be obtained. Since the diaphragm is in direct contact with the rod supporting the displaced plate, energy balance between the speaker and moving plate can be used to express the root-mean-square of plate velocity as follows:

$\frac{1}{2}m_{d}\cdot\dot{x}_{rms}^{2}=\frac{1}{2}m_{t}\dot{S}_{rms}^{2}+m_{t}\cdot g\cdot\Delta h-F_{d}\cdot S_{rms}$ (A7)

here: $m_{d}$ is mass of diaphragm, $m_{t}$ is total mass of rod and plate, $g$ is acceleration due to gravity, $\Delta h$ is distance between plate and diaphragm, $F_{d}$ is drag force and $S_{rms}$ is plate displacement. Since a sinusoidal wave is used, the amplitude of plate displacement can be expressed as: $S_{max}=S_{rms}\cdot\sqrt{2}$. Now, consider a loose particle motion on a plate shown in Figs. A1a and 1Ab.

|   x  δ  δ  n  τ | δ  δ  ${w+D}_{\tau}$  $F_{i}=ma_{i}$  $F_{f}$  ${w\cos\theta+D}_{n}{+F}_{ad}$ |
| --- | --- |
| **a)** | **b)** |

**Figure A1.** (**a**) A particle residing on glass surface, (**b**) Free-body diagram of a particle at onset of release

y

After assuming the equilibrium in $\tau$ and $n$-axes, the sum of forces acting on the particle at the onset of release can be expressed as:

| $\sum F_{\tau}=ma_{\tau}=-\frac{C_{D}\rho v_{R\tau}^{2}A}{2}-\mu_{f}mg\cos\delta$ | (A8) |
| --- | --- |
| and |  |

| $\sum F_{n}=ma_{Rn}=-\frac{C_{D}\rho v_{Rn}^{2}A}{2}-F_{ad}-mg$ | (A9) |
| --- | --- |
| It yields: |  |

| $a_{R\tau}=\frac{d^{2}{h\tau}}{dt^{2}}=g\sin\delta-\frac{C_{D}\rho\left( \frac{d\tau}{dt} \right)^{2}A}{2m}-\mu_{f}g\cos\delta$ | (A10) |
| --- | --- |
| and |  |
| $a_{Rn}=\frac{d^{2}h_{n}}{dt^{2}}=-\frac{C_{D}\rho\left( \frac{dn}{dt} \right)^{2}A}{2m}-\frac{F_{ad}}{m}-g\cos\delta$ | (A11) |
|  |  |

where: $F_{i}$ is the inertia force, $w$ is the weight, $D$ is the drag force, $A$ is particle cross sectional area, $F$ is the friction force, $N$ is the normal (reaction) force, $F_{ad}$ is the adhesion force, and *δ* is the inclination angle of the surface. The resultant displacement of particle from the surface is $h=\sqrt{h_{\tau}^{2}+h_{n}^{2}}$. Since the flow is air around the dust particle occurs at very low Reynold number (Re), Stokes’s law can be adopted in the drag terms shown in Equations (A10) and (A11). Then, the acceleration of the particle can be expressed as:

$\frac{d^{2}h_{\tau}}{dt^{2}}=gsin\delta-\mu_{f}gcos\delta-\frac{3\pi\mu D}{m}\frac{dh_{\tau}}{dt}$ (A12)

and

$\frac{d^{2}h_{n}}{dt^{2}}=-gcos\delta-\frac{F_{ad}}{m}-\frac{3\pi\mu D}{m}\frac{dh_{n}}{dt}$ (A13)

The solution to the second-order ODEs (i.e. Equations (A12) and (A13)) can be obtained by summing the complementary and particular solution. The final form of resulting displacement of the particle is:

$h_{\tau}(t)=\frac{mgsin\delta-\mu_{f}mgcos\delta}{3\pi\mu D}t-\frac{C_{1}{me}^{-\frac{3\pi\mu D}{m}t}}{3\pi\mu D}+C_{2}$ (A14)

$h_{n}(t)=\frac{-mgcos\delta-F_{ad}}{3\pi\mu D}t-\frac{C_{3}me^{-\frac{3\pi\mu D}{m}t}}{3\pi\mu D}+C_{4}$ (A15)

The expression for the vertical and horizontal velocities are:

$v_{\tau}(t)=\frac{mgsin\delta-\mu_{f}mgcos\delta}{3\pi\mu D}-C_{1}{me}^{-\frac{3\pi\mu D}{m}t}$ (A16)

$v_{n}(t)=\frac{-mgcos\delta-F_{ad}}{3\pi\mu D}-C_{3}me^{-\frac{3\pi\mu D}{m}t}$ (A17)

Incorporating the initial conditions that $\tau=0$, $n$= 0, $v_{\tau}=v_{1}$ and $v_{n}=v_{2}$ at $t=0$, the constants can be expressed as:

$C_{1}=\frac{\left( gsin\delta-\mu_{f}gcos\delta\right)m}{3\pi\mu D}-v_{1}$ (A18)

$C_{2}=\frac{C_{1}m}{3\pi\mu D}$ (A19)

$C_{3}=\frac{\left( -gcos\delta-\frac{F_{ad}}{m} \right)m}{3\pi\mu D}-v_{2}$ (A20)

$C_{4}=\frac{C_{3}m}{3\pi\mu D}$ (A21)

In reality, the friction term has very small influence on the kinetics of the repelling particles.

Therefore, by neglecting the frictional force terms, one has:

$h_{\tau}(t)=\left( \frac{-m^{2}gsin\delta}{\left( 3\pi\mu D \right)^{2}}+\frac{v_{1}m}{3\pi\mu D} \right)\left( 1-e^{-\frac{3\pi\mu D}{m}t} \right)+\frac{mgsin\delta}{3\pi\mu D}t$ (A22)

$h_{n}(t)=\left( \frac{m^{2}gcos\delta+F_{ad}m}{\left( 3\pi\mu D \right)^{2}}+\frac{v_{2}m}{3\pi\mu D} \right)\left( 1-e^{-\frac{3\pi\mu D}{m}t} \right)+\frac{mgcos\delta+F_{ad}}{3\pi\mu D}t$ (A23)

$v_{\tau}(t)={\frac{mgsin\delta}{3\pi\mu D}+\left( \frac{-m^{2}gsin\delta}{3\pi\mu D}+v_{1} \right)e}^{-\frac{3\pi\mu D}{m}t}$ (A24)

$v_{n}(t)=\frac{\left( mg+F_{ad}cos\delta\right)}{3\pi\mu D}+\left( \frac{m^{2}gcos\delta+F_{ad}m}{3\pi\mu D}+v_{2} \right)e^{-\frac{3\pi\mu D}{m}t}$ (A25)

v_1_ and v_2_ are the plate velocities, which are obtained from the experiments during the excitation of the plate from the sonic coil. A computer program is developed and Table A1 is used simulating the equations for the cluster of 1.732 mm dust cluster (in line with the experiment).

| **Parameter** | **Value** |
| --- | --- |
| Density of air, $\rho\left( \frac{kg}{m^{3}} \right)$ | 1.29 |
| Density of particle, $\rho_{p} \left( \frac{kg}{m^{3}} \right)$ | 2880 |
| Particle diameter, $D$ (*mm*) | 1.732 |
| Mass of particle (mg) | 0.0313 |
| Angle of inclination, $\theta$ | 0° - 45° |
| Coefficient of friction, $\mu$ (*Pa.s*) | 0.0404 |
| Adhesion force, $F_{ad}$ (*N*) | 2×10^-11^ |

**Table A1.** Parameters used in the simulation of equations.

**References**

1. Robert-H Munnig Schmidt. *Low Frequency Sound Generation by Loudspeaker Drivers*. *RMS Acoustics & Mechatronics* (2017).
